# The Collector Hypothesis: Who Benefits More from Art, the Artist or the Collector?

**DOI:** 10.1007/s12110-024-09481-7

**Published:** 2024-11-15

**Authors:** Piotr Sorokowski, Jerzy Luty, Wojciech Małecki, Craig S. Roberts, Marta Kowal, Stephen Davies

**Affiliations:** 1 Institute of Psychology, University of Wrocław, Dawida 1, Wrocław, 50-527 Poland; 2 Department of Philology, University of Wrocław, Wrocław, Poland; 3 Department of Psychology, University of Stirling, Stirling, UK; 4 Being Human Lab, Institute of Psychology, University of Wrocław, Wrocław, Poland; 5 Philosophy Department, University of Auckland, Auckland, New Zealand

**Keywords:** Collector hypothesis, Art collecting, Papuan art, Evolutionary theory, Fitness indicators

## Abstract

Human fascination with art has deep evolutionary roots, yet its role remains a puzzle for evolutionary theory. Although its widespread presence across cultures suggests a potential adaptive function, determining its evolutionary origins requires more comprehensive evidence beyond mere universality or assumed survival benefits. This paper introduces and tests the Collector Hypothesis, which suggests that artworks serve as indicators of collectors’ surplus wealth and social status, offering greater benefits to collectors than to artists in mating and reproductive contexts. Our study among Indigenous Papuan communities provides preliminary support for the Collector Hypothesis, indicating that, compared to artists, collectors are perceived as having higher social status and greater attractiveness to women. These findings provide unique insights into Papuan communities and contribute to the ongoing discussion about art’s adaptive significance of art by suggesting that artistic capacities may benefit not only creators but also those who accumulate and display art. Further research in diverse cultural contexts is needed for a comprehensive understanding of this interplay.

The enduring human fascination with art has deep historical and evolutionary roots. Archaeological findings provide insights into the early origins of art and craftsmanship, showcasing items like engraved shells and meticulously crafted hand-axes created by *Homo erectus* around 400,000–500,000 years ago (Joordens et al., 2014; Matthen, 2015). Neanderthals, too, left their artistic mark, evident in various forms of art, including cave paintings dating back 64,000 years (Clottes, 2010). Early Homo sapiens artistic expressions further enrich this evolutionary narrative, with the oldest known engraving and drawing discovered in South Africa (Henshilwood et al., 2002, 2018), illustrating how traces of artistic expression span historical periods and cultures globally—from the Arctic landscapes of the Inuit to the lush forests of Papua and the remote Pacific islands (Fraser, 1962). Notably, the oldest figurative image and hunting scene, both from Indonesia and dating back over 40,000 years, highlight the long-standing artistic tradition in the region that includes the Pacific islands studied here (Aubert et al., 2019; Brumm et al., 2021). Moreover, contemporary research in developmental psychology highlights the propensity of even young children to engage in artistic endeavors, such as composing songs and creating visual art, suggesting an intrinsic inclination toward creative expression (Yadav & Chakraborty, 2017). This collection of evidence suggests that artistic expression might be an evolved trait of our species.

However, at the same time, art poses a major challenge for evolutionary theory (Davies, 2012; Richards, 2019; Tooby & Cosmides, 2001). On the one hand, it is as universal across human cultures as it is costly, which suggests it is an adaptation (Dissanayake, 2019; Dutton, 2009). On the other hand, there is no clear theoretical consensus on this issue. Some theories propose that art has potential survival benefits, such as acting as a cognitive simulator or fostering social cohesion (Boyd, 2005; Gottschall, 2012; May et al., 2020; Sorokowski et al., 2024). Some others suggest that artistic capacities may also have sexual value, enhancing mating attraction, mate retention, or competition for mates (see below). Art would then be a reproductive adaptation rather than a survival one (Darwin, 1871; Miller, 2011; Zahavi & Zahavi, 1999).

Looking from the reproductive perspective, art is analogous to the decorations that male bowerbirds use to adorn their nests (Rothenberg, 2012). This analogy highlights important evolutionary processes, such as convergence (Currie, 2012; McGhee, 2011). While the decorations themselves have no direct survival value for the males, they function as an extended phenotype, signaling their fitness and aiding in attracting females (Kelley & Endler, 2012). Consequently, the trait of nest decoration is sexually selected for within the species (Driscoll, 2006; Miller, 2011). Similarly, while artistic works like paintings and sculptures may lack immediate practical use, they demand ingenuity, dexterity, and creativity—traits that can signal fitness (for a broader understanding of the phenomenon and other theoretical approaches, see Davis & Arnocky, 2022; Prum, 2012; Verpooten & Nelissen, 2012). In human contexts, artistic displays may influence reproductive outcomes, especially given that humans (probably) engage in more complex social dynamics than many other species. Unlike the pronounced sexual dimorphism observed in birds, humans typically exhibit higher paternal investment, with mutual mate choice playing a significant role (Walter et al., 2020). Therefore, females may also derive benefits from artistic displays, as evidenced by research indicating that creative expression can be linked to reproductive strategies (Stewart-Williams & Thomas, 2013; Varella et al., 2017).

It is perhaps surprising that, despite many publications in this area (e.g., DeLecce et al., 2022; Karamihalev, 2013; Miller, 2001, 2011; Novaes & Natividade, 2023; Varella et al., 2022; Voland & Grammer, 2003), there is a lack of research explicitly testing the adaptive role of practicing art and its impact on an artist’s potential reproductive success. Nonetheless, there is limited evidence that suggests (at least in Western communities) that more creative individuals or artists have more sexual partners (Beaussart et al., 2012; Clegg et al., 2011; Lange & Euler, 2014) and that women perceive artistically talented men (not necessarily artists) as more desirable partners (Clegg et al., 2008; Kaufman et al., 2016; Varella et al., 2022). As mentioned, this phenomenon is not straightforward, as some findings yield contradictory results. For example, Lebuda and colleagues (2021) showed that in one of the Papuan tribes, more creative men (it was not studied whether they were artists) had fewer children and fewer partners, while other studies conducted in Western societies found that musicians’ profiles were not rated as more attractive than non-musicians (Bongard et al., 2019). Perhaps the most intriguing study was conducted by Wassiliwizky et al. (2023). When participants were asked at the end of the study to explicitly compare artistic and non-artistic individuals, they favored the artists on almost all scales, including attractiveness. However, when the study compared an artist with a person of a different profession (which seems a more ecologically valid task, as this is what happens in real social settings), such as a farmer, teacher, physician, waiter, craftsman, or mechanic, this effect disappeared, and the musician was rated lower than most other professions. Furthermore, analyses of twin modeling in a large sample (over 10,000 twins) revealed no significant relationships between musical ability and measures of mating success. However, there was a very small association (*r* = 0.09) between the number of children and musical achievements in men (Mosing et al., 2015).

In this paper, we introduce the Collector Hypothesis as a new perspective on the relationship between art and reproductive theory. It posits that artworks serve as indicators of collectors’ surplus wealth and social status, offering greater benefits to collectors than to artists in mating and reproductive contexts. We would like to emphasize that this hypothesis complements previous research and findings in the field and does not contradict them. While artistic skill may signal certain aspects of fitness that could confer indirect benefits to a mate, it has been argued that it does not provide a clear signal of direct benefits, such as parental care or territory defense. Skills like hunting or farming, which can indicate both direct and indirect benefits, might offer more obvious advantages in mate choice. However, it is important to recognize that human mating strategies are multifaceted. Artistic skills may still play a role in sexual selection, particularly in contexts where traits such as creativity and social prestige enhance an individual’s desirability and success in attracting mates. On the other hand, as we also see in modern times, many artists are proverbially incapable of securing a stable, let alone prosperous, livelihood (Abbing, 2008). In the context of the above argument, but also the data described in the previous paragraph, it is not clear to what extent being an artist provides an advantage in sexual selection.

The answer we would like to suggest is related to the fact that, historically, those artists who thrive can often do so only because they are recognized and supported by those who already live particularly prosperous lives (Hauser, 1999). These would be kings, princes, barons, chiefs, merchants, middle-class citizens, and others who act as collectors—broadly understood—either simply buying works from artists, commissioning those works, or engaging in patronage (Culotta, 2017; Lord & Lord, 2010). There is ample evidence supporting this claim from late prehistorical times, including in ancient Egypt (Davis, 1983, 2020) and the Roman and Byzantine Empires (Gold, 2012; Hilsdale, 2014), the Middle Ages (Hourihane, 2013), the Renaissance (Burke, 2004; Hollingsworth, 2014; Lloyd, 2022; McLean, 2007), and onward through the age of Enlightenment (Ziskin, 2012), Romanticism (Castilho & Bennett, 1988), and Modernism (Hook, 2021) to the present day. We hypothesize that rare and costly artifacts and artworks function as a marker of collectors’ surplus wealth and, therefore, their capacity to provide direct benefits to prospective mates. It is important to clarify that, in this context, we are referring specifically to the accumulation of extraordinary artistic pieces as a marker of social success, rather than art as a general behavioral pattern or universal psychological capacity. If the possession of valuable artworks is a marker of social success, wealth, and status, it tends to benefit collectors more than artists themselves. Such a hypothesis is consistent with both the concept of honest (costly) signaling of wealth and resource-holding power, as well as a signal of good taste for high culture associated with the upper class, indicating status and reputation (Barker et al., 2019). In this study, we take a step toward testing this hypothesis by comparing the perceived attractiveness of artists versus collectors.

Our objective was to test these predictions through an empirical investigation among indigenous populations—the Asmat and Kamoro communities of Papua—renowned for their artistic achievements, particularly in sculpting (Rockefeller & Gerbrands, 1967; van der Zee, 2009). We took a preliminary step toward confirming our hypothesis by comparing the perceived social status, number of children, and attractiveness of artists and collectors to women. These affiliated societies utilize the Asmat-Kamrau Bay languages (Usher & Suter, 2020) and reside in remarkably similar ecological circumstances. Their renowned carved items encompass ceremonial spirit poles (*bjis* in Asmat, *mbitoro* in Kamoro), human and animal figurines, adorned shields, embellished musical instruments, decorated canoes, and ornate tools. What is crucial from the point of view of the present study is that both these communities have been producing art since long before their initial encounters with Westerners (Rockefeller & Gerbrands, 1967), and the artist role is not inherited, as is the case, for instance, with being a *griot* in West Africa (Kaschula, 1999).

## Method

### Participants

**Fig. 1:**
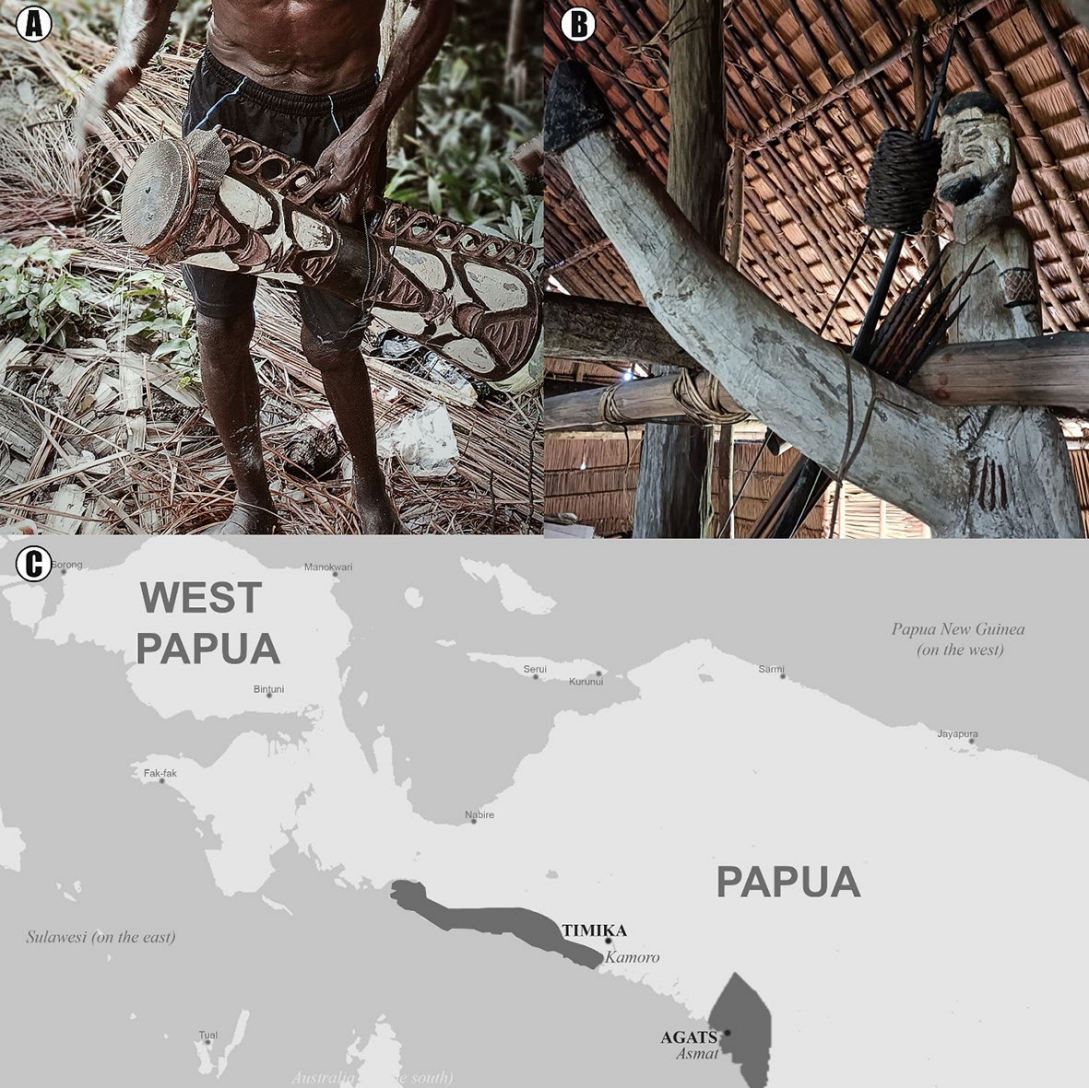
Map showing the regions where the study was conducted (**C**), with examples of artistic products in Asmat and Kamoro societies: (**A**) musical instrument, (**B**) sculptures inside “men’s house”

We recruited 180 individuals (76 women) from the Asmat and Kamoro region peoples, indigenous inhabitants of Papua (Indonesian province). The study was conducted near the villages of Kamora and Agats (e.g., in Kamora, Espe, Uus, Per, Amborep, and Warse). The locations of the mentioned societies and examples of their artistic products are shown in Fig. 1. The self-assessed age of participants ranged from 20 to 75 years old. Participants were compensated for their participation (equivalent to about 5 USD). The study was conducted following the recommendations of the Declaration of Helsinki. The Institutional Ethics Committee at the University of Wroclaw approved (2020/HBMIO) the study’s protocols and gave ethical approval for conducting the study. The local community leaders also approved the study.

### Procedure

As with all our previous studies, participants were informed that we wanted to have a brief conversation and gather their opinions. We explained to potential participants that the discussion would focus on the art created by people in their communities Participants were presented with the following task. After providing the information that “we expect their opinion and would like to hear their observations” we described the following scenario: “Two Papuan men were of the same age. Both men possessed numerous carvings. One man was a woodcarver responsible for creating these sculptures, while the other man was an individual who owned such sculptures—he had a large number of them.” How he acquired them was unspecified, but it was emphasized that he did not carve them himself; instead, it was noted that “he possessed many.” This latter man was referred to as the “collector.” In our study, we worked with a Papuan assistant, a man in his 40s, who conducted the interviews and asked the same set of questions to each participant. The idea of a collector was understood by our interviewees; they pointed out individuals who own many pieces of art, even though they do not create them themselves, and they grasped the concept of selling art to someone. At first, our intention was to carry out this study exclusively among women and inquire about their preferences. Nevertheless, due to significant interest expressed by many men in participating, we included similar questions for them. This allowed us to gather their perspectives on the mating success of both the artist and the collector.

Women were asked the following questions:

1. “If you were to choose a husband or were a young woman choosing a husband again, whom would you choose between them? — a woodcarver or a collector.”
2. “Which man, the woodcarver or the collector, has a higher social status (more important in the village)?”

Men were asked the following questions:

1. “Which man, the woodcarver or the collector, has a higher social status (more important in the village)?”
2. “Which man, the woodcarver or the collector, is more successful with women?”
3. “Which man, the woodcarver or the collector, has more children?”

## Results

55 of 76 women (72%; χ^2^ = 15.2, *p* < 0.001) favored the collector as a long-term romantic partner over the woodcarver. Simultaneously, 65 of 76 women (86%, χ^2^ = 38.4, *p* < 0.001) believed the collector had higher social status than the wood carver. Of 104 men, 99 (95%) indicated that the collector had a higher social status than the woodcarver (χ^2^ = 85.0, *p* < 0.001). Moreover, 101 out of 104 men (97%) suggested that the collector was more successful with women than the woodcarver (χ^2^ = 92.4, *p* < 0.001). However, only 54 of 104 men (52%) indicated that the collector had more children than the woodcarver, which was not statistically significant (χ^2^ = 0.2, *p* = 0.70). Furthermore, age was unrelated to the perceptions of collectors and artists on any of the questions (women assessing long-term romantic partner, χ^2^ = 0.01, *p* = 0.94; women assessing social status, χ^2^ = 0.17, *p* = 0.68; men assessing social status, χ^2^ = 0.27, *p* = 0.61; men assessing success with women, χ^2^ = 0.02, *p* = 0.89; men assessing the number of children, χ^2^ = 0.57, *p* = 0.45). Raw data from this study can be found at https://osf.io/s5xr6/?view_only=5dddb14567d348c2b31788b86c52b715.

## Discussion

In this study, we aimed to explore the potential reproductive advantages associated with the roles of artists and collectors within the context of indigenous Papuan communities. Our investigation was driven by the Collector Hypothesis, which suggests that artistic skill may function as a marker of collectors’ surplus wealth and, therefore, their ability to confer greater mating and reproductive benefits upon mates than the artists themselves.

Our results provide preliminary support for the Collector Hypothesis. Most surveyed women favored the collector over the woodcarver as a long-term romantic partner. Additionally, most women believed the collector had a higher social status than the artist. This observation could potentially explain women’s preference for collectors over artists. These findings bolster the idea that collectors, who possess a substantial collection of artworks created by artists, are perceived as more attractive and socially successful within these indigenous communities than the artists themselves. Although collectors may be more preferred in studied population, it doesn’t imply that artists have no sexual or reproductive success in Papua or other communities in the world. When selecting mates, individuals prioritize necessities first and then consider luxuries. In indigenous communities, where resources are scarce, people might prioritize indicators of resource acquisition over aesthetic creativity (see Li et al., 2002). The overwhelming majority of participants indicated that the collector had a higher social status than the woodcarver. Furthermore, almost all our participants suggested that the collector was more successful with women. These findings align with the notion that possessing art may serve as a signal of social success and status, making collectors more attractive and preferred partners in reproductive contexts. However, we did not observe any relationship between perceptions of having more children and the attributed status of an artist or a collector. This result can be explained in several ways. It is possible that artists are more preferred for short-term relationships, which we did not assess and which would lead to a similar number of offspring. This would align with the notion that resources are beneficial for child-rearing, while good genes are more relevant for short term sexual attraction (Buss, 2016). Another factor could be the typically high fertility rates in the Papuan population (Sorokowski et al., 2013), which might reduce the informativeness of the comparison. Future research could explore child mortality rates or the number of wives of artists and collectors as potentially more meaningful indicators.

At a speculative and theoretical level, it is worth highlighting that our findings do not necessarily contradict prior research suggesting that individuals with artistic or creative skills might be perceived as desirable partners (Beaussart et al., 2012; Clegg et al., 2011; Miller, 2011). Instead, our data points to an alternative pathway: while being an artist is one way to increase one’s attractiveness, accumulating art as a collector might signal status and provide direct benefits to potential partners. This suggests that the role of art in enhancing partner desirability can operate through multiple mechanisms, with collectors potentially using their amassed art as a unique signal of social status. While this idea is compelling, it remains outside the scope of our current analysis and calls for further research.

Our findings raise important questions regarding the role of artistic skills in sexual selection. While some prior literature, including Miller’s (2011) earlier work, has suggested that artistic abilities are viewed positively in the context of mate selection, our data indicate a preference for collectors over artists. This does not negate previous findings but rather suggests that artistic skills are one of many factors influencing mate attractiveness and that collecting might increase mate attractiveness to a greater degree It is essential to consider that art may serve as a means of livelihood for artists, allowing them to generate income that can be offered as direct benefits to potential mates. In this context, artistic abilities could be viewed as contributing to overall attractiveness, but not as the sole determinant of mate choice. Furthermore, we acknowledge that artistic capacities may indicate a variety of traits, such as intelligence and creativity, which can be appealing in the mate selection process. Our study presents a nuanced perspective, suggesting that while artistic talents may enhance an individual’s appeal, they are part of a broader social and economic framework that influences mate selection.

We recognize the need for further investigation into the dynamics of artistic skills and their implications for sexual selection. Future research could explore how these various elements interact and coexist in different cultural contexts, including the potential for mutual mate choice and the influence of economic factors in artistic production. Collecting behaviors entail substantial costs in terms of effort, money, time, and space needed to find, obtain, and store desirable items. The non-utilitarian nature of collectibles suggests that a simple economic justification for this behavior is unlikely. Notably, the apparent sex difference, with collectors being almost exclusively men, indicates a possible reproductive motive. Our research aligns with previous analyses on collecting, examples of which are presented below.

Apostolou (2011) argues that collecting has evolved to facilitate reliable communication between males regarding their unobserved resource acquisition capacity. This framework posits that the desirability of a collectible item is positively related to its rarity, aesthetic pleasantness, and size. The evidence from eBay auction sales of fossilized dinosaur eggs supports these hypotheses, highlighting how collecting can signal an individual’s status and resources. In addition, a recent summary by Koliofotis (2022) draws upon ideas from sexual selection and costly signaling theory, arguing that conspicuous consumption has evolved as a sexually selected mating strategy. Koliofotis outlines criteria for identifying traits that are outcomes of sexual selection and explores the idea of conspicuous consumption as a form of sexual adaptation. While acknowledging the contributions of evolutionary theory to understanding consumption behavior, he critiques existing explanations for not adequately considering human evolved psychology and evidence from past environments. He proposes that cultural evolution theory offers an alternative explanation, viewing conspicuous consumption as a behavior marked by specific social learning mechanisms. This approach highlights the cognitive factors involved in consumption choices, linking it to the broader context of resource signaling. Finally, Danet and Katriel (1994) present a conceptual analysis of the elements of play and aesthetics in collecting. The main focus is on the process of collecting as a form of human experience among both children and adults. Drawing on materials from popular literature on collecting and interviews with about 165 adult and child collectors in Israel, the paper analyzes how objects become collectibles and the aesthetic principles that guide the construction of a collection. The central hypothesis posits that collecting is a means to strive for a sense of closure, completion, or perfection. Finally, our research well complements previous research projects that discussed, in an evolutionary context, the mechanisms of male collecting behavior (Apostolou, 2011; Lee et al., 2022; see also the example of birds: Doerr, 2012) and the mechanisms of sexual selection of conspicuous consumption (Koliofotis, 2022). Our research shows that men’s art collecting may influence their success with the opposite sex.

As to how far back can the human tendency to collect art can be traced, one fascinating clue comes from the Makapansgat cobble, a small dark red jasperite pebble dating almost 3 million years, discovered in a cave inhabited by Australopithecines. Though unmodified, its natural markings resemble a face, and it had been carried at least 32 km to the cave. This suggests that early hominins were captivated by its facial appearance, possibly appreciating the resemblance. As Bednarik (1998) suggests, this could indicate an early form of aesthetic recognition. This discovery implies that artistic cognition might have emerged from a general appreciation of beauty, eventually leading to the collection of objects with suggestive forms, such as the proto-figurines from Tan-Tan (300,000–500,000 years ago) and Berekhat Ram (250,000–280,000 years ago), which may have been made by *Homo heidelbergensis* (Bednarik, 2003) While this evidence highlights early aesthetic awareness, it raises questions about the role of collecting behaviors in prehistoric societies. Among most hunter-gatherer groups, individuals typically do not possess significantly more wealth or objects than others, although some may attain higher prestige (Marlowe, 2010). Thus, it is unlikely that collectors or patrons were present within such societies, suggesting that the collector hypothesis may not fully account for the origins of art or the proliferation of artistic activities during the Upper Paleolithic period and earlier (Henshilwood et al., 2002). Aesthetically pleasing objects might have been collected solely for their beauty. However, the population described in this study, like other populations in Papua (e.g., members of the Yali tribe exhibit large differences in the number of pigs owned; Sorokowski et al., 2013), does not demonstrate the same degree of egalitarianism seen in previous and contemporary hunter-gatherer societies. This variability in wealth and status may have influenced early collecting behaviors and the development of artistic appreciation.

Itis also crucial to acknowledge the limitations of our study. First, we would like to clarify that our analysis is limited to portable artifacts, such as sculptures or paintings. Forms of art such as music, theater, poetry, etc., typically do not yield collectible works of art. Second, our investigation focused on indigenous Papuan communities, raising questions about the generalizability of our findings to other cultural and social contexts. Future studies could encompass a more diverse range of populations and utilize additional methodologies to better understand the intricate relationship between art, social status, mate choices (including those for short- and long-term), and reproductive success. Nonetheless, we deliberately selected our community to investigate its evolutionary past, presuming it may offer a better insight into a bygone period compared to contemporary Western societies, and we chose a traditional society known for its artistic achievements (van der Zee, 2009). Also, our procedure did not specify that the artist and collector had a set number of carvings (e.g., 12 pieces). Instead, it emphasized that both possessed “numerous” carvings, which could have led participants to assume that the collector had more carvings than the artist. Finally, this study, like many others conducted in Western societies (e.g., Bongard et al., 2019; Wassiliwizky et al., 2023), focuses on perceptions and preferences rather than on actual sexual selection and its consequences (e.g. reproductive success).

In conclusion, our study provides novel evidence that supports the Collector Hypothesis, suggesting that individuals who accumulate and possess artworks created by others—known as collectors— may potentially enjoy greater sexual benefit of long term relationships in terms of social status and desirability. The study provides new insights into the potential evolutionary role of collecting behaviors in sexual selection, emphasizing the significance of collectors in the relationship between aesthetic appreciation, status, and reproductive success.

## Data Availability

The data can be accessed at https://osf.io/s5xr6/?view_only=5dddb14567d348c2b31788b86c52b715.
